# Liquid Marble Actuator for Microfluidic Logic Systems

**DOI:** 10.1038/s41598-018-32540-w

**Published:** 2018-09-20

**Authors:** Thomas C. Draper, Claire Fullarton, Neil Phillips, Ben P. J. de Lacy Costello, Andrew Adamatzky

**Affiliations:** 1 Unconventional Computing Laboratory, University of the West of England, Bristol, BS16 1QY UK; 2 Institute of Biosensing Technology, Centre for Research in Biosciences, University of the West of England, Bristol, BS16 1QY UK

## Abstract

A mechanical flip-flop actuator has been developed that allows for the facile re-routing and distribution of liquid marbles (LMs) in digital microfluidic devices. Shaped loosely like a triangle, the actuating switch pivots from one bistable position to another, being actuated by the very low mass and momentum of a LM rolling under gravity (~4 × 10^−6^ kg ms^−1^). The actuator was laser-cut from cast acrylic, held on a PTFE coated pivot, and used a PTFE washer. Due to the rocking motion of the switch, sequential LMs are distributed along different channels, allowing for sequential LMs to traverse parallel paths. This distributing effect can be easily cascaded, for example to evenly divide sequential LMs down four different paths. This lightweight, cheap and versatile actuator has been demonstrated in the design and construction of a LM-operated mechanical multiplication device — establishing its effectiveness. The actuator can be operated solely by gravity, giving it potential use in point-of-care devices in low resource areas.

## Introduction

Liquid Marbles (LMs) are small droplets of liquid that have been coated in a nano- or micro-powder^1^. Also known (less theatrically) as particle-coated droplets, the liquid is typically aqueous and the powder coating has a degree of hydrophobicity — resulting in non-wetting of said powder. Due to the hydrophobic nature of this powder, the minimal energy profile of the water droplet results in a near-spherical coated droplet, that does not wet hydrophilic surfaces. Whilst an aqueous droplet with a hydrophobic particle coating is by far the most common form of a LM, there are also examples using an organic liquid core and oleophobic coating^2^.

This phenomenon results in the ability to easily transport microlitre quantities of liquid around, with zero loss due to surface adhering/wetting. The advantages to microfluidics are obvious^3^, and are increased by the high-mobility and variability of LM manipulation: controlled movement of LMs has been demonstrated using lasers^4^, magnets^5^, electrostatic-forces^6^, the Marangoni effect^7^, and gravity^1^. Another well-documented use of LMs is as miniature chemical-reactors^8^. By encapsulating one’s reaction in a LM, the reaction is conducted quickly, cheaply and with great ease of parallelisation (allowing for potential use in high-throughput screening).

The practical uses of LMs are plentiful. Due to the steadily increasing widespread interest in LMs^9–14^, there are reports of their use in a number of fields. For example, LMs have been used as micro-incubators for the viable growth of mammalian embryonic stem cells^15^; for rapid blood-typing assays by injection of antibodies into a “blood-marble”^16^; and as signals in mechanical collision-based computation^17^.

Previously reported computation with LMs has utilised a conservative logic collision-based approach in the construction of an interaction gate^17^. In this, the Boolean value true was portrayed by the presence of a LM and false was portrayed by the absence of a LM. By having two LMs approaching each other from different directions, computation would be performed once the LMs collide and resultantly change direction; conversely if there is only one LM, then there is no collision and no change in direction. In a pure collision-based computer the LMs can move about in free space, momentarily ‘creating wires’ as required. This, combined with the ease of tuning a LM’s core and coating, allows for an elegant system.

This collision-based system, however, requires the precise synchronisation of the data signals (i.e. the LMs). Whilst this has been achieved with the use of magnetic LMs and synchronised electromagnets, it places an additional burden on the experimental computational setup. As such we have developed a sequential mechanical computing device, based on our new LM-actuated flip-flop switch, to demonstrate the new flip-flop actuator. In our design the data signals are represented by LMs, and the result of the computation is displayed in the positioning of the bi-stable flip-flop actuators.

These new switches are able to be mechanically actuated by the very low mass and momentum (~4 × 10^−6^ kg ms^−1^) of a LM. By having a reproducibly activated flip-flop switch, it is possible to cascade them in series, forming circuits and even computation.

Such a system also has great scope as a digital microfluidic platform^18,19^. In digital microfluidics, discrete droplets can operate as virtual reaction chambers. In these chambers reactions are carried out on a micro-scale, minimising the use of reagents and cost. Current digital microfluidic use includes point-of-care diagnostics^20^ and ad hoc synthesis of hazardous materials^21^. Our actuator allows for the facile distribution of sequential LMs into different parallel paths.

There are many methods of actuating droplets in digital microfluidics, with the most common being electrowetting on dielectric (EWOD)^18^, magnetic^20^, and surface acoustic wave (SAW)^22^. These techniques all have advantages and disadvantages, which have been discussed in-depth elsewhere^23^. However, they all share two major disadvantages. Firstly, they require the surface to be pretreated to make it hydrophobic (at a minimum, there are often other surface requirements) — preparing a high-quality low-surface-energy substrate is both expensive and critical to traditional digital microfluidic devices. Secondly, they all require electricity. This second point may seem strange, but many microfluidic point-of-care devices are used in resource-poor environments, where the electrical supply can be unreliable and even batteries can be a luxury^24^.

Our device manipulates discrete microlitre droplets of water, through a system of switches and pathways, while negating these two negative points of EWOD, magnetic and SAW actuation: our surface treatment is quick, cheap and easy (being applied with a commercial aerosol); and the device does not require electricity to actuate the LMs. This is achieved by encapsulating the droplets in a hydrophobic powder (i.e. forming LMs), and allowing them to roll through a series of ramps, bridges and switches under gravity. By pre-setting the switches, a series of LMs can be controlled through different pathways, thus allowing different LMs to undergo different processing, when combined with more traditional microfluidic techniques. By joining microfluidics with mechanical computing, a new dimension of processing can be accessed. A simple example of this is a system that counts the number of LMs that have passed through the device. More elaborate designs can conduct calculations, such as multiplication — discussed below and in the Electronic Supplementary Information (ESI).

## Materials and Design

In order to demonstrate our flip-flop actuating switch we decided to pair microfluidics with unconventional computation, in the design and construction of a digital microfluidic platform with mathematics as its primary function. Our model was inspired by original ideas of toy-like mechanical computers proposed in mid 1960s: computer-type device^25^, game machine employing multi-state swinging gates^26^, and binary digital computer^27^ (which was further modified into the educational mechanical computer Digi-Comp II). All photographs were taken using a Nikon Coolpix P90 camera; and the videos were recorded with a Nikon Coolpix P90 and an fps1000HD-256 camera (Imagetec Ltd).

The device was designed in Autodesk AutoCAD 2018 and manufactured by laser-cutting the parts out of 3 mm thick clear cast acrylic. The separate parts were then temporarily held in position by clamps or pins, before being affixed together using RS Pro AB-3 Liquid Acrylic Adhesive (RS Components), forming the backboard. This is then sprayed with a cheap copper coating (Kontakt Chemie EMI 35, Farnell element14), in order to reduce static build-up. Without this, the static built-up by some LMs hindered reliability by causing them to adhere to the surface. The spray does impart a slight texture to the acrylic, though this does not noticeably affect the rolling of the LMs. Due to the joining of two 3 mm sheets, the dimensions of the backboard are 306 mm × 409 mm × 6 mm.

**Figure 1:**
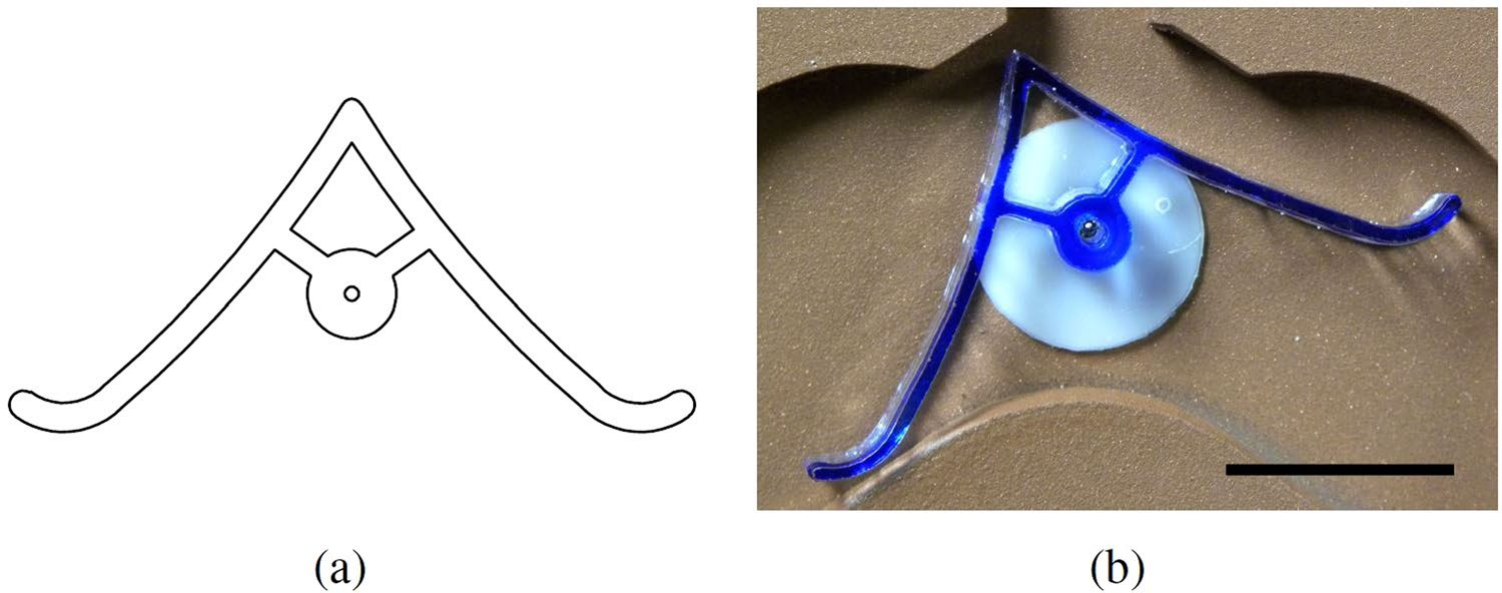
(**a**) A diagram and (**b**) *in-situ* photograph of the LM-actuated flip-flop actuator. The scale bar is 10 mm.

The flip-flop actuators were designed and constructed in the same manner as the main device. A CAD drawing and a photograph of the flip-flop can be seen in parts (a) and (b) of Fig. 1, respectively. The CAD file is available in the ESI. The switches are bi-stable, resting towards the left and right directions, and as such can indicate binary 0 or binary 1. They have been designed such that the centre of mass is longitudinally and latitudinally centred, positioned at the pivot point. The dimensions of the switch are 28 mm × 13 mm × 3 mm, with a mass of 160 mg.

The large automatic LM formation ramp at the top was made hydrophobic using a commercial hydrophobic spray (Rust-Oleum^®^ NeverWet^®^). The indent was then filled with powdered ultra-high density polyethylene (PE) (Sigma-Aldrich, 3–6 × 10^6^ g mol^−1^, grain size approximately 100 μm).

Polyethylene was used because recent work has suggested that LMs made with PE have a higher resistance to impacts than other tested coatings^28^. Unfortunately, PE LMs build up static on acrylic much faster than our Ni-PE hybrid LMs used previously^17^, ergo the previously mentioned copper spray coating.

Flip-flop switches were attached to the acrylic backboard by means of a pivot, which was placed in a pre-cut hole and glued in position. The pivot was composed of a 0.25 mm pin, with PTFE insulation, giving an outer diameter of 0.50 mm. A PTFE washer (OD = 10.0 mm, ID = 0.6 mm, 0.25 mm thick) was then slotted onto each pivot, followed by a flip-flop switch. The PTFE washer was laser-cut in house from sheet PTFE.

The device was pitched at an angle of 54.8° from horizontal. Water droplets (17.8 ± 0.1 μL) were permitted to fall at the top of the ramp and run over the powder bed, generating LMs. These *in situ* formed LMs roll into the top of the calculating device under gravity, pass through the device, and finally exit at either the bottom-left or bottom-right corner.

Water droplets were provided through an 18G, standard bevel, vertically aligned, stainless steel needle. A constant flow was provided through the needle via syringe pump. In order to ascertain the average droplet size, 20 droplets were dropped onto an analytic balance (Ohaus Adventurer AR0640) and their masses measured. The resulting masses, the average mass and standard deviation can be seen in Table S1, ESI.

All data generated or analysed during this study are included in this published article (and its supplementary information files).

## Results

### Microfluidic Mechanical Multiplier

**Figure 2:**
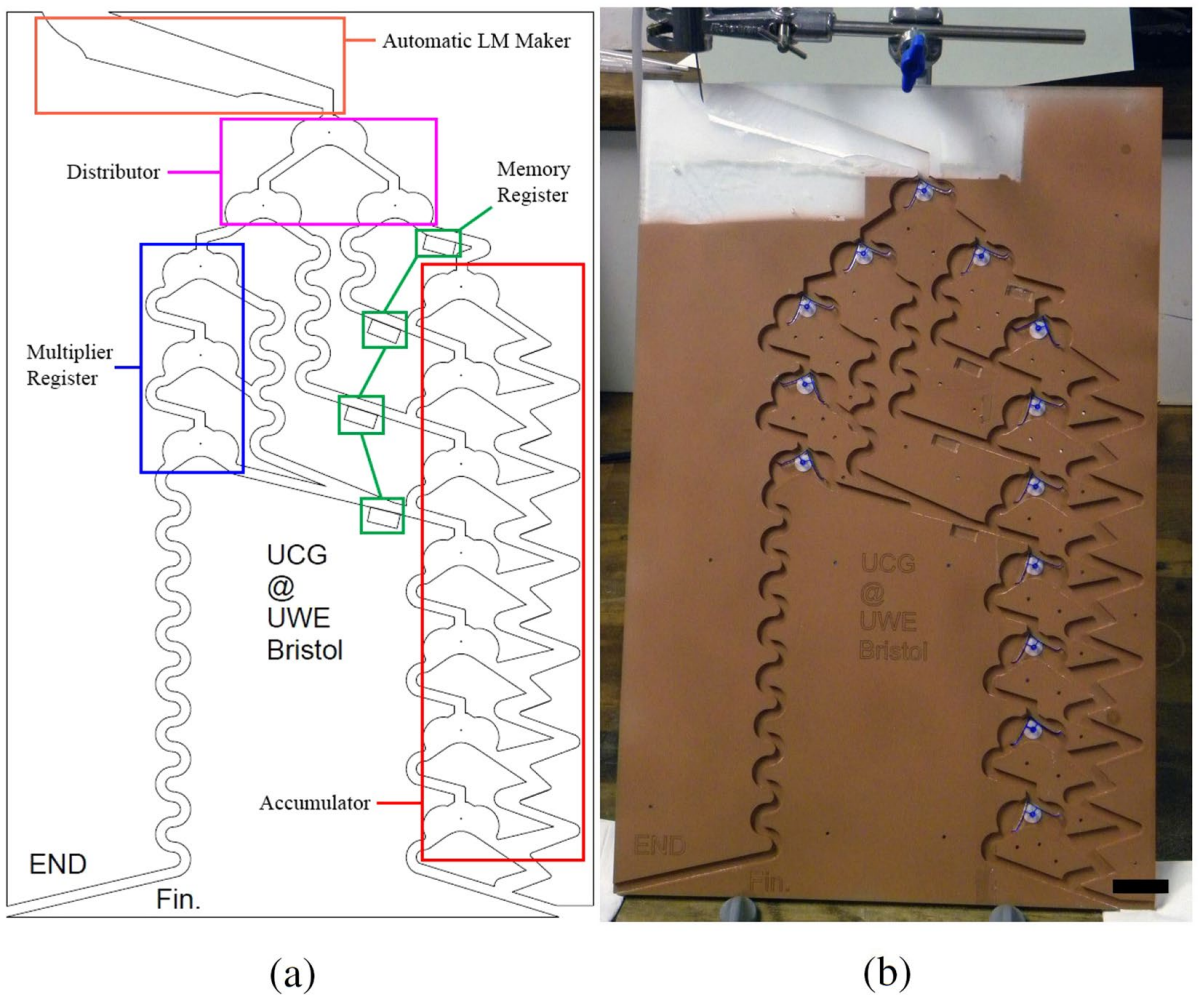
(**a**) A colour-coded diagram of the LM multiplying device. The sections are as follows: orange is the automatic LM maker, purple is the distributor, blue is the multiplier register, green is the memory register, and red is the accumulator register. (**b**) A photograph of the fully constructed LM multiplying device. The scale bar is 3 cm.

The mechanical computer is comprised of many flip-flop switches and some bridges, creating five main sections — each labelled with a different colour in Fig. 2(a). The sections are the automatic LM maker (orange), the distributor (purple), the multiplier register (blue), the memory register (green), and the accumulator register (red). These will each be discussed below. A photograph of the fully constructed device can be seen in Fig. 2(b).

The flip-flop actuators, visible in Fig. 1, are small and lightweight switches that can be reliably actuated by the low mass of a LM — approximately 18 mg in this case. This low actuating force meant designing such a switch was intractable. The flip-flops are bistable, and as such can rest in one of two positions: with the peak of the triangle pointing at either −23° or +23° from vertical. This is used to indicate binary 0 or binary 1. In this way, the switches act like traditional electronic flip-flops. On encountering a flip-flop switch, the mass of a LM causes rotation of the switch around the pivot, thereby changing the value. At the pinnacle of rotation, the LM is released (along a pathway determined by the new position of the switch) and the switch remains in the new resting position. This results in sequential LMs being routed through alternating pathways.

**Figure 3:**
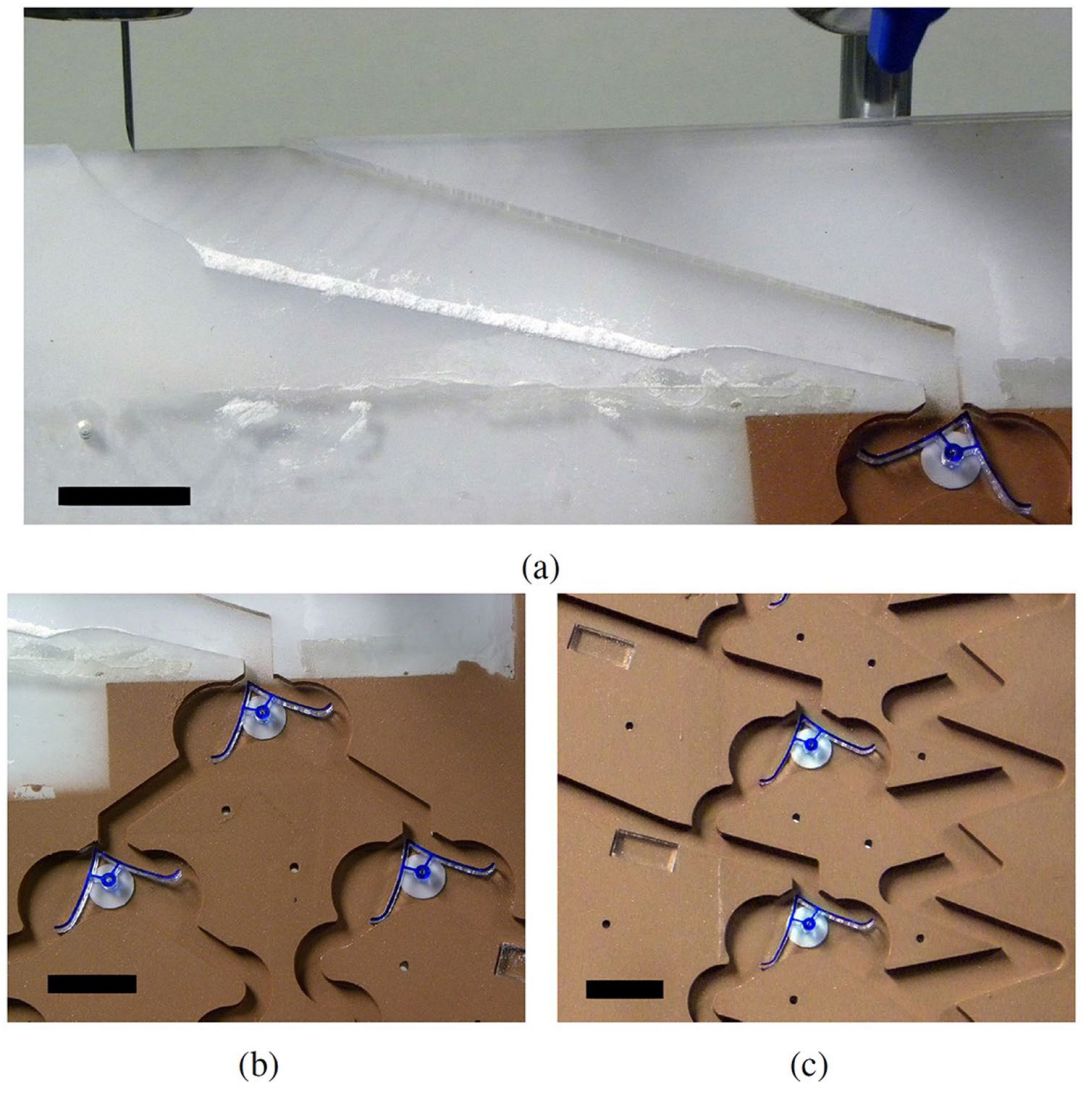
Up close photographs of (**a**) the automatic marble maker, (**b**) the distributor, and (**c**) sections of both the memory and accumulator registers. All scale bars are 2 cm.

The automatic LM maker (orange in Fig. 2(a) and shown in part (a) of Fig. 3) was first exampled in our work on collision-based computing^17^. A water droplet of known volume is permitted to fall onto a superhydrophobic surface, before rolling over a hydrophobic powder bed. This forms a LM, which is then permitted to enter the distributor. A steady stream of consistently sized water droplets can be produced by using a syringe pump and needle.

The distributor (purple in Fig. 2(a) and shown in part (b) of Fig. 3) is comprised of three flip-flops. It’s function is to evenly distribute successive input LMs to the four outputs. In order for the LMs to follow the required pathway, they should all be pointing to the right at the start of any calculation.

The multiplier register (blue in Fig. 2(a)) is also comprised of three flip-flops. By pointing the flip-flops to the left or right, they can be made to indicate binary 1 or 0, respectively. As such the multiplier register can hold any three digit binary number (i.e. 0 to 7 in decimal), read from the bottom up. The device multiplies by serial addition, and the number held in the multiplier register at any moment is the number of addition operations remaining.

The memory register (green in Fig. 2(a) and partly visible in part (c) of Fig. 3) is unique on the device, in that it is not comprised of flip-flops. Instead, four bridging-blocks are used to store a four bit binary number (i.e. 0 to 15 in decimal), read from the bottom up. Binary 1 is indicated by the presence of a bridging-block, and binary 0 is indicated by the absence of such a block. As a LM approaches the memory register, if it encounters a 0 it is stopped by the missing bridge, while if it encounters a 1 it continues along the bridge into the accumulator register.

The accumulator register (red in Fig. 2(a) and also partly visible in part (c) of Fig. 3) is comprised of seven flip-flop switches, able to store a seven bit binary number (i.e. 0 to 127 decimal), read from the bottom up. These switches indicate binary 0 in the left position, and binary 1 in the right position, contrary to the multiplier register. The accumulator, as its name implies, accepts input binary numbers and accumulates them, adding them to the previously stored number. This is conducted by the pulses of LMs. If a LM enters at the first flip-flop then decimal 1 is added to the accumulator, if a LM enters at the second flip-flop then decimal 2 is added to the accumulator, if a LM enters at the third flip-flop then decimal 4 is added to the accumulator, and if a LM enters at the fourth flip-flop then decimal 8 is added to the accumulator. On reaching a flip-flop, the LM queries if it currently indicates binary 0 or 1; if it is binary 0 then it flips it to binary 1 and the LM leaves the system; if it is binary 1 then it flips it to binary 0, and then moves down to query the next flip-flop switch.

By combining these features the mechanical multiplier can sequentially add, i.e. multiply, up to a limit of 7 × 15 = 105. This limit is set by the size of the device and total number of flip-flop switches, and is purely practical in nature — there is theoretically no limit to the complexity of calculation that can be performed with an appropriately sized device.

It is also possible to use the device for a singular addition, with an upper limit of 15 + 112 = 127. This is achieved by setting the multiplier register to decimal 1, and placing the two summands in the memory register and accumulator register. This, in effect, results in the mechanical multiplier adding one lot of the memory register to the contents of the accumulator register.

Examples of both multiplication and addition operations are described in detail in section S2 of the ESI. The number of LMs required for a particular operation, and their pathways, are also chronicled there. The pathway that a LM would take, with a pre-determined flip-flop arrangement, is graphically portrayed in Fig. S1 of the ESI as a visual aid.

### Liquid Marble Dynamics

**Figure 4:**
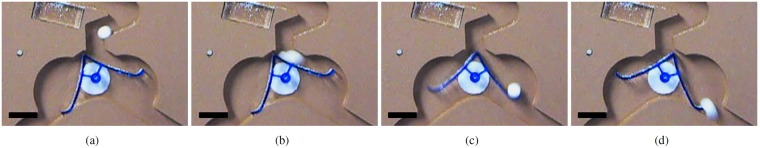
Frames taken from a video demonstrating the actuating of our flip-flop switch by a LM. Relative frame times are (**a**) 0 ms, (**b**) 100 ms, (**c**) 300 ms and (**d**) 500 ms. All scale bars are 10 mm.

Video footage was recorded of the PE LMs actuating the flip-flop switches. Still frames were extracted, and can be seen in Fig. 4.

It can be seen that the LMs navigate the flip-flop switch in approximately 500 ms. This was consistent across the device, due to the ‘resetting’ of the LMs velocity at every flip-flop. The incline of the device means that LMs accelerate after each encounter with a flip-flop, preventing them from loosing enough momentum to fail actuation of a switch. On a straight run, a LM can build up sufficient speed to destroy itself on impact with a solid wall. For this reason we designed into the device both alternating ramps and separate twisting sections, visible in Figs 2 and 3(c). These were successful at preventing premature LM destruction.

Small amounts of the PE powder coating were visible leaving the LMs and adhering to the surface of the device. These small amounts only built up to a certain level (visible in Fig. 4), and are a culmination of many tiny losses from multiple LMs. It is believed that once a small powder coating is deposited on the surface, the slightly loose nature of the newly powdered surface prevented any further depositions. As a result, the lost powder does not build-up sufficiently to block any channel, and the LMs who have lost some powder are still able to roll freely.

A frame-by-frame analysis of the video footage was conducted. From this, it was possible to determine a speed for a LM as it moved through the device, by measuring the distance it travelled in a certain number of frames, and knowing the frame timestamps. We calculated a typical speed of 0.22 ms^−1^, which compares well with previously reported speeds for non-coalescing, impact-surviving LMs^6,17,29^. Impact velocity of LMs hitting the side wall at particular points of the system can be seen in Table 1. It should be noted that no toroidal or peanut-shaped LMs are observed. This is because the velocity of our LMs is much lower than that reported for such deformations (approximately 1 ms^−1^)^1^.

**Table 1:** Speeds of the LMs as they move through the system.

LM Location	Speed/ms^−1^
Accumulator Entrance	0.22
Accumulator Exit	0.15
LM Maker Exit	0.26
Distributor Internal	0.24
Average (SD)	0.22 (0.04)

The average speed and its standard deviation (SD) is also shown. Speeds were calculated by measuring the distance a LM had travelled in a known number of frames.

## Discussion

Used as a digital microfluidic platform, this gravity-powered mechanical flip-flop actuator provides many advantages. Firstly is the ability for the flip-flop to evenly distribute sequential LMs into alternate pathways. By arranging an additional flip-flop on each exit path (as demonstrated in Fig. 3(b)), sequential LMs are evenly divided between four paths. An additional layer of flip-flops allows for even distribution amongst eight paths, and so on. Secondly, it can operate with zero electrical power (the main system uses no power, and the syringe pump is easily replaced by a syringe droplet), making it attractive for situations such as point-of-care diagnostics in developing countries. A potential point-of-care use of our actuator is in the loop-mediated isothermal amplification (LAMP) of DNA. In a digital microfluidic device reported by Wan *et al*. multiple consecutive droplets are separated into different regions using EWOD^30^. These consecutive droplets could be easily distributed into the required four separate zones using our flip-flop switches. A similar use could be implemented in the digital microfluidic polymerase chain reaction (PCR) device reported by Wulff-Burchfield *et al*., with EWOD-powered droplet separation replaced with electricity-free flip-flop switches^31^. Thirdly, LMs are able to encapsulate the aqueous buffer-conditions often required for bioassays, as well as more arduous environments. And fourthly, as the LM moves through the system it is rolling, as oppose to the sliding motion often observed in microfluidics. This imparts a level of mixing to the contents of the LM, which is often highly desirable.

In our view, the additional physical features are not discourteous to the ideals of microfluidics. Indeed, many existing microfluidic platforms already instigate physical barriers; such as for pathway restriction, or to physically halt a discrete droplet for dispensing or particle-removal purposes^20,32,33^. Indeed, the straightforward mechanical nature of the flip-flop means that the switch is highly stable and durable (a repetition video is available in the ESI). Being non-reliant on electricity, it is also resistant to some of the failures of other manipulation methods.

The frame-by-frame video analysis revealed that there were two different, but equally effective, techniques for actuating the flip-flop switch via a LM. The first technique is visible in Fig. 4: the LM enters the flip-flop, rolls down to the extremity of the arm, causing a rotation of the switch, and thereby release of the LM. The second technique became more prevalent if the LM entered the actuator at speed: the LM enters the flip-flop by rebounding off its top point, it then moves quickly along the arm and collides with the upwards-pointing tip of the arm, this impact causes the actuator to start rotation, meanwhile the LM squeezes between the arm and the side-wall and continues along the device *before* the flip-flop has completed its rotation.

A high-speed video of a LM actuating a flip-flop switch was recorded using an fps1000HD-256 camera (Imagetec Ltd). The video was filmed at 1000 fps with a resolution of 1280 × 720, playback was performed at 25 fps for an effective slowdown of 40x. This video, available in the ESI, demonstrated that a combination of the two actuating techniques was sometimes used, also with great effect. Also apparent in this video is the comparatively violent nature of the LMs path. At every impact, collision or change of direction the stresses on the LM cause it to warp in shape. A common side-effect of this is the loss of particle coating from the LM. It should be noted that whilst this loss of coating is very small, it is not negligible, and therefore will eventually amount to destroy the LM by wetting of the surface. This reinforces the notion of LM lifetime^6,13,28,34^.

The applied torque (τ_app_) is the actual torque applied to the flip-flop switch by the LM. It can be calculated using Eq. (1), where *r* is the distance between the mass and the rotation centre (8.5 mm), *F* is the force acting on the flip-flop (18  mg × 9.81 ms^−2^ = 1.77 × 10^−4^ N) and *θ* is the angle between the force and the lever arm. In this case, the lever arm and applied force are in line and so the only influence on *θ* is the tilt of the device (54.8°). This gives a value of applied torque of 1.23 × 10^−6^ Nm.

1 $${\tau }_{{\rm{app}}}=rF\,\sin \,\theta $$

The moment of inertia of the flip-flop switch can be calculated using Eq. (2), where *I* is the moment of inertia, *m* is the mass of the object, and *k* is the radius of gyration. The radius of gyration was determined as 6.76 mm, using Autodesk AutoCAD 2018. This gives a moment of inertia of 7.31 × 10^−9^ kg m^2^.

2 $$I=m{k}^{2}$$

The net torque (τ_net_) is the resulting torque on the system, after frictional effects. Knowing the moment of inertia, it is possible to calculate the net torque using Eq. (3). Here, *α* is the angular acceleration of the flip-flop switch. This was determined from the high-speed video to be 152 rad s^−2^. The resulting net torque is 1.11 × 10^−6^ Nm. The frictional effects of the system can be quantitatively determined as the friction torque (τ_F_). This value can be calculated using Eq. (4), as the ‘missing torque’ between applied and net torque values. In our system, the friction torque was 1.20 × 10^−7^ Nm.

3 $${\tau }_{{\rm{net}}}=I\alpha $$

4 $${\tau }_{{\rm{net}}}={\tau }_{{\rm{app}}}+{\tau }_{{\rm{F}}}$$

The total work done (*W*_tot_) by a LM actuating a single flip-flop can be described using Eq. (5), where *θ*_rot_ is the total rotation of the flip-flop in radians. The the flipping of the switch from −23° to +23° represents a total rotation of 0.80 rad. Using the values calculated for torque, the total work performed is 9.84 × 10^−7^ J.

5 $${W}_{{\rm{tot}}}={\tau }_{{\rm{app}}}{\theta }_{{\rm{rot}}}$$

From this, it can be said that the speed of the LM entering the flip-flop is not a determining feature. As long as the LM has entered the flip-flop, it will actuate the switch using its gravitational potential energy, before exiting and rolling downhill to the next flip-flop. What is significant is the weight of the LM, as described by *F* in Eq. (1) — which must be large enough to have sufficient torque to rotate the flip-flop.

A curious observation was made when two LMs were permitted to roll next to each other. When the two LMs make contact, they do not roll together like smooth ball-bearings. Instead, they bounce off each other. This is because the rear of the leading LM is moving upwards, whilst the front of the approaching LM is moving downwards. When the two opposite directing motions meet they clash, resulting in the chasing LM rolling backwards briefly. This is caused by the rough texture of the LM surface.

An interesting feature of these flip-flops, is that they do not fully actuate if the LM is too small. Instead, the small LM moves along and around the flip-flop, leaving it in the same position. This phenomenon could be used for sorting LMs. If a string of small LMs approaches the flip-flop they will all exit along the same path, until a large LM approaches and actuates the flip-flop, after which the small LMs will exit along the other path, until a large LM approaches and actuates the switch again.

By designing a system to operate on LMs, the evaporation rate of the droplets is reduced^28^. Therefore, it is not necessary to cover the droplets with oil to prevent evaporation, as is often the case with more traditional microfluidic systems; or to form a phospholipid layer around the droplets^35^. There are many advantages to this: heating the sample requires much less energy, and so can be done quicker; there is no risk of cross-contamination from reagents diffusing through the oil^20^; and costs are kept down, by both the reduced energy use as well as the decrease in oil/solvent use.

A possible future development of the multiplication device would be to investigate the use of liquid metal marbles (LMMs)^36^. Due to the powder coating inherently present on a LMM, the high surface tension of the liquid metal (e.g. Galinstan) would not be an advantage directly — however it would enable the use of a wider range of powders. The high cohesive forces within liquid metals results in a very high surface tension (534.6 mN m^−1^)^37^, and minimal adhesive forces between both the powder coating and the device surface. This means that LMMs would be less prone to premature rupture or or accidental surface wetting. Indeed, the wettability of liquid metals has already been shown to be tunable^38^. The greater density of the liquid metal core would also be advantageous in actuating the flip-flop switches. Additionally, initial investigations towards the regular formation of liquid metal droplets — important for the automatic creation of LMMs — has been reported by others already^39^.

## Conclusions

Here we have reported on a new routing device for LMs, in the form of an actuating flip-flop switch. This flip-flop has been designed to be lightweight, easy to use, and cheap to manufacture. By optimising the design of the flip-flop and length of the arms, using PTFE washers and a PTFE-coated pivot, we are able to actuate the flip-flop switch with the very low mass and momentum of a LM.

Further studies in this field could look at the miniaturisation of the flip-flop switch. Certainly LMs can be made much smaller, however our access to manufacturing techniques restricted the size we could make the flip-flop. However, other materials and manufacturing techniques could be used to make the flip-flop smaller, thereby allowing use of even smaller LMs.

It is believed that LMs have a strong future in microfluidics. However for their full use to be realised, an increased toolbox for behavioural control needs to be developed. Typical tools required will include auto-generation, routing, merging and dividing of LMs. This new flip-flop design presents a new routing technique for LMs, not before seen in microfluidics. Its ability to alternate directions of sequential LMs will prove useful.

In a digital microfluidic system, the swing of the flip-flop arms and its bistable positions results in sequential LMs being directed into different alternate directions. This novel distributing phenomenon can be cascaded, allowing one input to be divided between 2, 4, 8 … outputs. An example of a 1-input 4-output arrangement is shown in Fig. 3(b) where sequential LMs can be dispensed equally between four distinct channels from one input channel.

The demonstration model of the LM-actuated mechanical multiplying device establishes the versatility of this flip-flop design. To the authors knowledge, this also represents the first time that LM digital microfluidics has been partnered with mechanical multiplication.

We envision that this flip-flop switch could be of use in point-of-care devices in low resource environments, such as for distributing droplets into separate zones for different reaction conditions. The gravity-powered nature of the design means that no electricity is required, helping to keep running-costs down. Additionally, the systems state is not lost or modified due to a lost of power/input (either electricity or LMs). Many traditional systems will loose their current setting and/or reset when power/input is resumed, for example a counter may reset back to zero. In our mechanical set-up this is not an issue — if the stream of LMs is interrupted for any reason then the system will remain unchanged indefinitely until another LM is introduced to the device.

## Electronic supplementary material

Supplementary Information

ESI Videos and CAD file
