# Supplementary material for: Liquid Marble Actuator for Microfluidic Logic Systems
Source: Sci Rep. 2018 Sep 20;8:14153. doi: 10.1038/s41598-018-32540-w (PMC6147806; doi:10.1038/s41598-018-32540-w)
Supplement: Supplementary file 1 — Supplementary Information [file 41598_2018_32540_MOESM1_ESM.pdf]

Electronic Supplementary Information

Liquid Marble Actuator for Microfluidic Logic Systems

Thomas C. Draper*[a], Claire Fullarton[a], Neil Phillips[a],
Ben P. J. de Lacy Costello[b], and Andrew Adamatzky[a]

[a]Unconventional Computing Laboratory, University of the West of England, Bristol, BS16 1QY, UK
[b]Institute of Biosensing Technology, Centre for Research in Biosciences, University of the West of England, Bristol, BS16 1QY, UK
*Corresponding author. Email: Tom.Draper@uwe.ac.uk

S1 Table of Water Droplet Sizes

Table S1: Water droplets provided by an 18G, standard bevel, vertically aligned, stainless steel needle. A steady water flow was provided by a syringe pump. Drops landed on a weighing boat, and were weighed on an analytical balance (Ohaus Adventurer AR0640). Standard deviation (SD) is shown in brackets.

	**Mass / mg**	
**Drops**	**Aggregate**	**Individual**
1	17.7	17.7
2	35.7	18.0
3	53.3	17.6
4	71.1	17.8
5	88.8	17.7
6	106.7	17.9
7	124.5	17.8
8	142.4	17.9
9	160.2	17.8
10	177.9	17.7
20	356.1	17.8
**Average (SD)**		17.8 (0.1)

S2 Example Operations

S2.1 Multiplication

In this example, we will multiply 2 by 3. This operation will take nine liquid marbles (LMs) to complete, and shall be discussed marble-by-marble. The starting set-up for such a calculation is as follows: the three distributors should be pointing to the right; the multiplier register should read binary 010 from the bottom up; the memory register should read binary 0011 from the bottom up; and the accumulator register should read binary 0000000. Throughout this section the bits shall be referred to using the Least Significant Bit as zero (LSB 0) convention (i.e. bits are numbered from right to left, starting at zero). The LMs perform

calculations on the mechanical calculator by following a simple set of rules. These are shown in table S2. Figure S1 examples a path taken by a LM.

Table S2: Logic rules to follow as the liquid marbles move through the mechanical multiplier, interacting with the various flip-flops switches and bridges.

Register	Physical Indicator	Bit	Action
Multiplier	Flip-Flop Left	1	Change bit to 0, continue onto memory register
	Flip-Flop Right	0	Change bit to 1, query next bit (or END if none)
Memory	Bridge Present	1	No change, continue to accumulator register
	Bridge Absent	0	No change, exit system
Accumulator	Flip-Flop Left	0	Change bit to 1, exit system
	Flip-Flop Right	1	Change bit to 0, query next bit (or OVERFLOW if none)

The first formed LM enters the distributor and exits along pathway 1, entering the multiplier register. The first flip-flop (bit 0) reads a 0, so it is changed to a 1 and the LM moves down to the next flip-flop. On querying this it reads a 1, so it changes it to a 0 and exits the multiplier register. The net effect is that the LM has read the multiplier register, subtracted one from it and rewritten it: thereby a destructive readout. The LM then enters the memory register at bit 3 (physically at the bottom). Here it reads a 0, in the form of a missing bridge, and the LM exits the system.

The second LM enters the distributor and exits along pathway 3, directly entering the memory register at bit 1. Here it reads a 1, and so exits the memory register. The LM has effectively read the memory register, and proceeded accordingly, without modifying the value stored: thereby a non-destructive readout. The LM then enters the accumulator register, also at bit 1. Here it queries the flip-flop and reads a 0, so it changes it to a 1, and then exits the system.

The third LM enters the distributor and exits along pathway 2, directly entering the memory register at bit 2. Here it reads a 0, and so exits the system.

The forth LM enters the distributor and exits along pathway 4, directly entering the memory register at bit 0. Here it reads a 1, and so passes on into the accumulator memory, also at bit 0. On querying the flip-flop switch the LM reads a 0, and so it changes it to a 1, and then exits the system.

At this point the device has completed one cycle, having effectively added 3 to the accumulator once. The next set of LMs effectively add a further 3, which will complete the sum 2×3.

The fifth LM enters the distributor and exits along pathway 1, entering the multiplier register. On querying the first flip-flop switch (bit 0) a readout of 1 is given, so the LM changes it to a 0 and exits the multiplier register. It then enters the memory register at bit 3, where it reads a 0, and so exits the system.

The sixth LM enters the distributor and exits along pathway 3. It enters the memory register at bit 1, where it reads a 1, and so passes on to enter the accumulator at bit 1. Here the flip-flop already reads 1 (from a previous LM), and so it is changed to a 0 before moving down to the next flip-flop switch (bit 2). Here it reads a 0, so it is changed to a 1, and the LM now exits the system.

The seventh LM enters the distributor and exits along pathway 2. It enters the memory register at bit 2, where it reads a 0, and therefore exits the system.

The eighth LM enters the distributor and exits along pathway 4. On entering the memory register at bit 0 it reads a 1, and so continues on to enter the accumulator register at bit 0. On querying the flip-flop switch it reads a 1 (set by a previous LM), and so it changes it to a 0 and moves down to bit 1. Here it reads a 0 (also set by a previous LM), so it changes it to a 1, and exits the system.

The ninth LM enters the distributor and exits along pathway 1. On querying bit 0 of the multiplier register it reads a 0, so it changes it to a 1, before moving down to bit 1. Bit 1 also reads as 0, so it changes it to a 1, before moving down to bit 2. Bit 2 also reads as 0, so it changes it to 1, before exiting the system along a unique path (bottom left of the device). This indicates the end of the calculation.

At this stage the accumulator register reads binary 0000110, or decimal 6, which is the correct answer to our calculation. Note that the ninth LM does not actually do any computing, it simply indicates that the calculation is complete by exiting along a special separate pathway.

S2.2 Addition Operations

Addition can be conducted in a similar manner to multiplication. In this case the multiplier register is set to 001, and the two summands are placed in the memory and accumulator registers. The device operates by sequentially adding the memory register to the accumulator register, with the number of iterations dictated by the multiplier register. Therefore, if the multiplier register reads one, then the device will add the memory register to the accumulator register, once: effectively performing simple addition.

Due to the accumulator register being larger than the multiplier register, it can handle larger numbers: the upper limit of addition is $15 + 112 = 127$ for this device. As a result of the systematic calculation procedure, the number of LMs required for any operation can be determined in advance using the equation $4M + 1$, where M is the number stored in the multiplier register at the start of a calculation. Because of this, any addition operation will always require exactly five LMs to complete.

S3 Example Path of a Liquid Marble

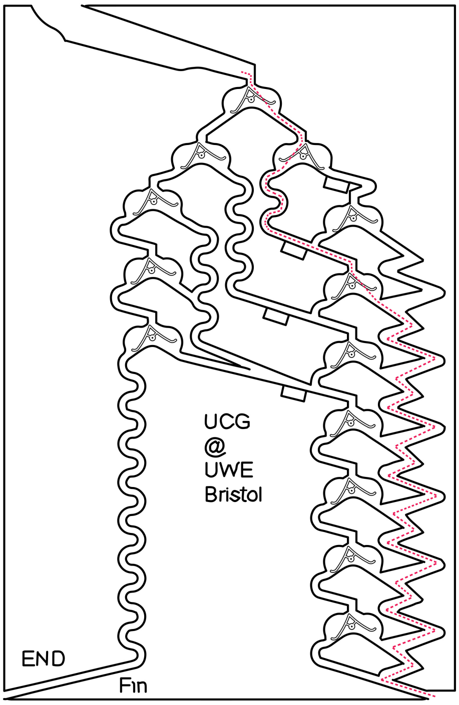

Figure S1: A schematic demonstrating the path a LM would take through the device, when the flip-flop switches are arranged as shown. The path is indicated by a red dotted line.

S4 Failure Modes of Pre-Optimised Models

Before the device was fully optimised, a peculiar and interesting failure mode was occasionally observed. If the gap between the top of the flip-flop switch and the boundary wall was too large, then the LM could pass through the gap. This is possible due to the flexibility of LMs. The majority of LMs did not take this path; and of those who did, only a small number survived. A photo of a failed attempt can in seen in figure S2. It should be noted that, in this case, the gap between the wall and flip-flop switch was only circa 1 mm.

Whilst considered a failure for this particular rigid mechanical system, it could equally be used as a design aspect for size exclusion or sorting in future circuits. Our design was inspired by a device that uses non-deformable rigid sphere, and so adaptation was required to incorporate the soft sphere properties of LMs. Further microfluidic platforms for LMs could factor their soft nature into the design from the beginning. An

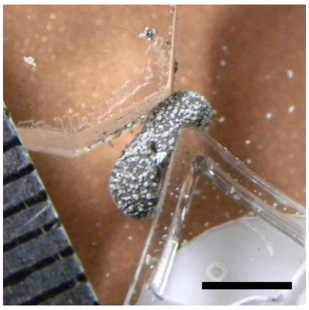

Figure S2: A photograph demonstrating a failure mode in an early model. The LM has come from the right, squeezed through the 1 mm gap between the wall and the flip-flop, before coming to a stop. The LM imaged has a Ni-PE coating. The scale bar is 3 mm.

example would be allowing consideration for the spherical shape of a static LM, versus the various deformed shapes LMs take under different velocity regimes.

Another issue that had to be overcome was the design and size of the flip-flop switch. In order for the switch to be actuated by a LM it had to be as light as possible, however there were other limiting factors to contend with. The thickness of the flip-flop arms, for example, could not be reliably laser-cut thinner than 1.0 mm using our equipment. The Gaussian-style laser beam could also create slanted edges and non-cylindrical pivot holes. The length of the flip-flop arms was also optimised empirically. A longer arm improves the mechanical advantage of the pivot, but also increases the moment of inertia of the flip-flop. It was necessary to find an optimal point between the extremes, which would allow for LM-actuated rotation. Schematics of two designs that were not able to be actuated using a LM can be seen in figure S3.

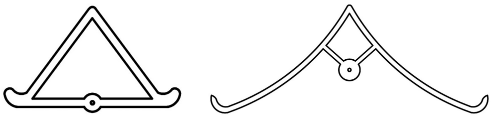

Figure S3: Schematics showing two early flip-flop designs. Neither of these designs were able to be actuated by a LM.

Friction (static and kinetic) is another force that had to be minimised. To help enable ease of rotation, the centre of mass of the flip-flop was always designed to be at the pivot point. In order to reduce the friction, we used PTFE-coated pivot-pins. This, combined with 0.25 mm PTFE washers, reduced the friction sufficiently to enable rotation.

S5 Other Supporting Files

Included in the electronic supplementary information (ESI) are videos portraying the flip-flop switch in action. A description of these videos can in found in table S3. The CAD file for the flip-flop is also included, as ‘flip-flop.dxf’.

Table S3: Descriptions of the videos included in the ESI.

File Name	Description
LM flip-flop 1000fps	A slow motion video of a LM passing through a flip-flop
Repeated Actuation	A single flip-flop being repeatedly actuated
